# Basic Concepts in G-Protein-Coupled Receptor Homo- and Heterodimerization

**DOI:** 10.1100/tsw.2007.197

**Published:** 2007-11-02

**Authors:** Rafael Franco, Vicent Casadó, Antoni Cortés, Carla Ferrada, Josefa Mallol, Amina Woods, Carme Lluis, Enric I. Canela, Sergi Ferré

**Affiliations:** ^1^ Institut d'Investigació Biomèca August Pi I Sunyer, Centro de Investigación Biomédica en Red Sobre Enfermedades Neurodegenerativas (CIBERNED), and Departament de Bioquímica i Biologia Molecular. Universitat de Barcelona. Diagonal 645, 08028 Barcelona, Catalonia, Spain; ^2^ National Institute on Drug Abuse, Intramural Research Program, National Institutes of Health, U.S. Department of Health and Human Services, Baltimore, MD 21224, USA

**Keywords:** receptor heterodimers, G-protein-coupled receptors, cooperativity, allosteric modulation, drug screening

## Abstract

Until recently, heptahelical G-protein-coupled receptors (GPCRs) were considered to be expressed as monomers on the cell surface of neuronal and non-neuronal cells. It is now becoming evident that this view must be overtly changed since these receptors can form homodimers, heterodimers, and higher-order oligomers on the plasma membrane. Here we discuss some of the basics and some new concepts of receptor homo- and heteromerization. Dimers-oligomers modify pharmacology, trafficking, and signaling of receptors. First of all, GPCR dimers must be considered as the main molecules that are targeted by neurotransmitters or by drugs. Thus, binding data must be fitted to dimer-based models. In these models, it is considered that the conformational changes transmitted within the dimer molecule lead to cooperativity. Cooperativity must be taken into account in the binding of agonists-antagonists-drugs and also in the binding of the so-called allosteric modulators. Cooperativity results from the intramolecular cross-talk in the homodimer. As an intramolecular cross-talk in the heterodimer, the binding of one neurotransmitter to one receptor often affects the binding of the second neurotransmitter to the partner receptor. Coactivation of the two receptors in a heterodimer can change completely the signaling pathway triggered by the neurotransmitter as well as the trafficking of the receptors. Heterodimer-specific drugs or dual drugs able to activate the two receptors in the heterodimer simultaneously emerge as novel and promising drugs for a variety of central nervous system (CNS) therapeutic applications.

## INTRODUCTION

The existence of neurotransmitter receptor homo- and heterodimers is becoming accepted, although with some initial resistance from the scientific community. This acceptance implies changing and broadening classical notions about neurotransmission. In fact, neurotransmitter receptors cannot be considered as single functional units, but as forming part of multimolecular aggregates localized in the plane of the plasma membrane, what are also called “horizontal molecular networks”[1,2,3].

It first became evident that G-protein-coupled receptors (GPCRs) are able to form receptor homodimers and also higher-order homomultimers. In fact, it seems that most, if not all, members of this neurotransmitter receptor superfamily can exist as homodimers[4,5,6]. As early as in the mid-1970s, radioligand binding experiments showed negative cooperative interactions among β-adrenergic receptor binding sites, similar to that previously described for receptor tyrosine kinases, such as receptors for insulin and nerve growth factor[7,8,9]. However, those results were interpreted as if the radioligand was binding to two different monomeric entities, i.e., the receptor uncoupled to G protein (displaying low affinity) and the receptor coupled to G protein (displaying high affinity). Then, biphasic curves obtained in binding isotherms or in competition experiments were usually interpreted in the frame of the so-called “two independent-site model”[10].

At the beginning of the 1980s, the existence of GPCR homodimers and -multimers was suggested for β-adrenergic and muscarinic acetylcholine receptors from experiments with photoaffinity labeling and immunoaffinity chromatography[11,12]. Subsequently, a great number of GPCRs were shown to form homodimers or -multimers by Western blotting, or by coimmunoprecipitation in transfected/infected cells or in samples from brain[1] and, more recently, by “resonance energy transfer” techniques in transfected living cells: fluorescence and bioluminescence resonance energy transfer (FRET and BRET, respectively)[4,5,6,13,14].

At the beginning of the 1980s, radioligand binding experiments showed the existence of neurotransmitter receptor-receptor interactions in brain membrane preparations. A first study demonstrated that the neuropeptide substance P modulates the high-affinity serotonin binding sites in spinal cord membrane preparations[15]. In these kind of interactions between different neurotransmitter receptors, known as “intramembrane receptor-receptor interactions”, stimulation of one receptor changes the binding characteristics of another receptor for endogenous or exogenous ligands in crude membrane preparations[1]. This implies the lack of involvement of intracellular signaling and suggests some kind of cooperative interaction between adjacent receptors. A great number of intramembrane receptor-receptor interactions involving GPCRs has been described[1] and, in 1993, following the discovery of neurotransmitter receptor homodimers, it was hypothesized that the molecular mechanism could be the formation of neurotransmitter receptor heterodimers[16]. In fact, in several cases, intramembrane receptor-receptor interactions have been shown to be a biochemical characteristic of receptor heterodimers, i.e., an intramolecular cross-talk within the dimer (see below). As for receptor homodimers, different techniques, including FRET and BRET, have demonstrated the existence of heterodimers in artificial cell systems[1,4,5,6]. The challenge consists of demonstrating their presence in the CNS.

## THE DIMER COOPERATIVITY INDEX

For GPCRs, the simplest molecule expressed at the cell surface is not the monomer, but the dimer (homo or hetero). Dimer-based models have been recently devised that allow fitting of data to receptor dimers[10,17,18,19] (Fig. 1). Assuming the simplest case, i.e., a homodimer radioligand binding to the receptor dimer requires a slightly more complex equation than the binding to a monomer, but this extra complexity gives statistically better results than the fitting to a model assuming the occurrence of two independent sites, one with high affinity and another with low affinity for the agonist. Importantly, models of ligand-receptor binding that consider the homodimer as the minimum structure for GPCRs are

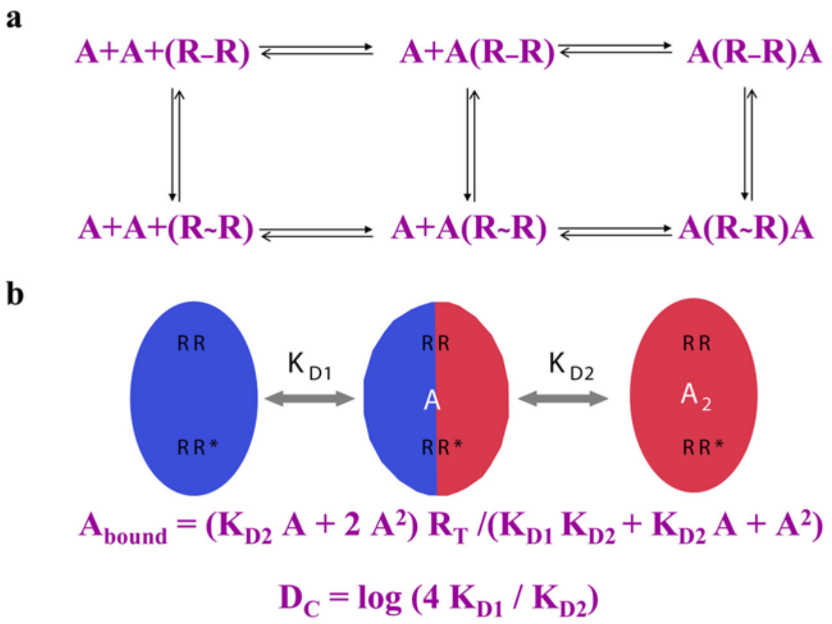

**FIGURE 1.** Scheme and equations of the two-state dimer model. (a) Model including all equilibria between inactive receptor dimers (RR), active receptor dimers (RR*), and the corresponding dimers with one or two ligand (A) molecules[18,19]. (b) Simplified model including macroscopic equilibrium dissociation constants ($K_{D1}$ and $K_{D2}$), and a simple equation for the ligand bound as a funtion of $R_T$ , $K_{D1}$, and $K_{D2}$, [A] and $[A]^2$. Binding data can be fitted to this simple equation using any nonlinear regression approach. The equation for calculating the dimer cooperativity index ($D_C$) is also displayed; $D_C$ can be readily obtained from $K_{D1}$ and $K_{D2}$ values[10].

able to predict both positive and negative cooperativity[10,17,19]. The existence of dimer molecules, as it occurs in the case of hemoglobin, which is a tetrameric molecule, makes possible an intramolecular cross-talk that is reflected as cooperativity. Hemoglobin shows positive cooperativity for the binding of oxygen, with each additional oxygen molecule binding "better" than the previous one[20]. Receptor dimers often display negative cooperativity, which is reflected by concave-upward Scatchard plots obtained from radioligand binding assays, although positive cooperativity has also been described for some GPCRs[10,18,19] (Fig. 2).

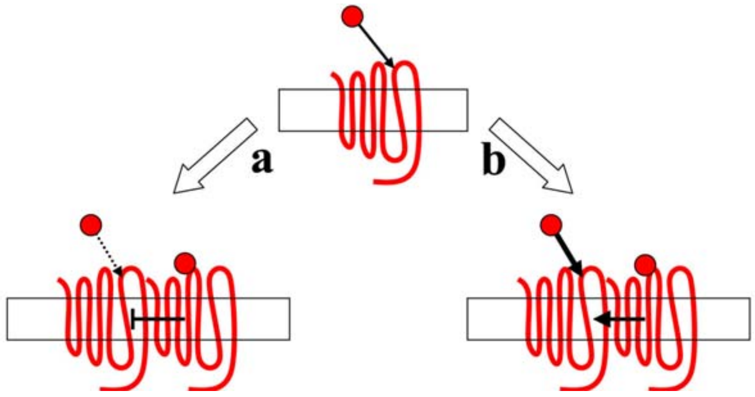

**FIGURE 2.** Intermolecular interactions in the receptor homodimer. The affinity of a given ligand for its receptor (central drawing) may be decreased (a) or increased (b) when the ligand already occupies the receptor partner (in the homodimer).

The receptor-dimer model devised by Franco et al.[10,18,19] allows quantification of cooperativity by defining a new parameter, the “dimer cooperativity index”[10] (Fig. 1). This index is very useful to know to what extent the binding of one neurotransmitter to one orthosteric center in the dimer is sensed in the second orthosteric center present in the partner receptor. Therefore, the cooperative index is a measure of the intermolecular cross-talk (intramolecular, if we consider the dimer as a molecule composed of two subunits) happening within the receptor dimer. In other words, the biphasic curves that often appear when performing binding assays reflect the intramolecular cooperativity within the dimer molecule. Mechanistically, this is explained by the transmission of the conformational changes caused to one receptor by ligand binding to the partner receptor. Cooperativity appears when these conformational changes modify the binding of the second ligand to the partner receptor. The binding of the second ligand can be “better” (positive cooperativity) or “worse” (negative cooperativity). The dimer cooperativity index is defined in a way that zero cooperativity index indicates lack of cooperativity, whereas a positive or negative value indicates positive and negative cooperativity, respectively[10].

The assumption of GPCR dimerization opens a new way to understand allosteric modulators, a variety of compounds or macromolecules that bind to the receptor to a nonorthosteric center, and affect their pharmacology and their physiological function[21]. The most paradigmatic example is the heterotrimeric G protein. This protein is known to bind to the intracellular loops of receptors that are therefore called GPCRs. In practice, though, the heterotrimeric G protein is an allosteric modulator of receptor dimers that can eventually modify the binding characteristics of the orthosteric centers in the dimer[10].

Variations in the binding characteristics due to the binding of small or big molecules to allosteric centers can be measured according to a receptor-dimer model in two ways. On one hand, an allosteric effector may change the affinity of the agonist(s) but, on the other hand, allosteric effectors may change the cooperativity index[10]. In a typical example, addition of GTP, which activates the heterotrimeric G protein, leads to the disappearance of cooperativity. In this example GTP will shift the cooperativity index to a zero value[10].

Among the large GPCR family, class C receptors represent a well-recognized example of constitutive dimers, both subunits being linked, in most cases, by a disulfide bridge[22]. The extracellular binding domains (the Venus flytrap domains, VFTs) can oscillate between an open and a closed conformation, the latter being stabilized by agonists. The relative orientation of the VFTs also oscillates between at least two positions, the resting “R” orientation, and the active “A” orientation, the latter being stabilized when at least one VFT is in a closed conformation, and further stabilized if both VFTs are closed. The heptahelical domains can also exist in at least three states, one responsible for the constitutive activity of some receptors, the fully inactive state stabilized by inverse agonist, and the fully active state stabilized by the active form of the dimer of VFTs[23]. Such complex cooperative functioning of these receptors offers a number of possibilities for allosterically regulating their activity using compounds acting at various sites of the receptor. One such possibility is to further stabilize the closed state of the VFT after agonist binding[23]. Allosteric modulation of metabotropic receptors is a novel way to pharmacologically manipulate GPCRs acting at a site that is distinct from the orthosteric binding region of the receptor protein. Allosteric modulators are thought to confer new pharmacological possibilities if compared with conventional agonists, as is the case of modulators that act at ligand-gated ion channels. Modulators of $GABA_A$ receptors are used therapeutically; for example, benzodiazepines amplify the action of the endogenous neurotransmitter GABA at the class C $GABA_A$ receptor. Allosteric-positive modulators of $GABA_B$ receptors, namely, CGP7930 and the more efficacious compound GS39783, have also been identified and characterized *in vitro*[24].

## TOOLS TO DEMONSTRATE THE OCCURRENCE OF RECEPTOR DIMERS IN THE CNS: THE DIMER FINGERPRINT

Although the identification of receptor dimers (homo and hetero) *in vitro* is well established, we are only at an early stage in the investigation of dimers *in situ*. One of the main concerns when a new homo- or heterodimer is described in an artificial cell system is its actual occurrence in natural tissues. In fact, FRET and BRET are mainly useful to detect receptor-receptor interaction in heterologous systems that are overexpressing the selected receptors. Then the question is how we can identify receptor dimers in the CNS. It is worth mentioning that arrays of dimers of the non-neurotransmitter GPCR rhodopsin have been visualized in disc membranes by using infrared-laser atomic-force microscopy[25]. Unfortunately, visualization of other GPCRs (not so abundant in a given cell) is not yet possible. Therefore, other approaches have been explored with success. A practical way is to find a "biochemical fingerprint", a biochemical characteristic displayed by the heterologous cell system where receptor dimerization has been demonstrated by means of, for instance, FRET and BRET techniques. A biochemical fingerprint of a receptor heterodimer can often be obtained with radioligand binding techniques. Thus, the affinity for a specific ligand can be modified when the receptor for that ligand is forming heterodimers (Fig. 3). As an example, the affinity of the adenosine $A_{2A}$ receptor for its natural antagonist caffeine is markedly reduced when it heteromerizes with the adenosine $A_1$ receptor[26]. A common biochemical fingerprint for receptor heterodimers is an intramembrane receptor-receptor interaction[1], which can only be attributed to the existence of an intermolecular interaction between the two receptor subunits (intramolecular, if we consider the heterodimer as one molecule composed of two subunits). In this case, the binding of a ligand (usually an agonist) to one receptor modifies the affinity of the other receptor for the same (in case of isoreceptors, i.e., different receptor subtypes for the same neurotransmitter) or another ligand (Figs. 3 and 4). For homodimers, cooperativity found by biphasic kinetics or nonlinear Scatchard plots in radioligand binding experiments can constitute a valid fingerprint (Fig. 2).

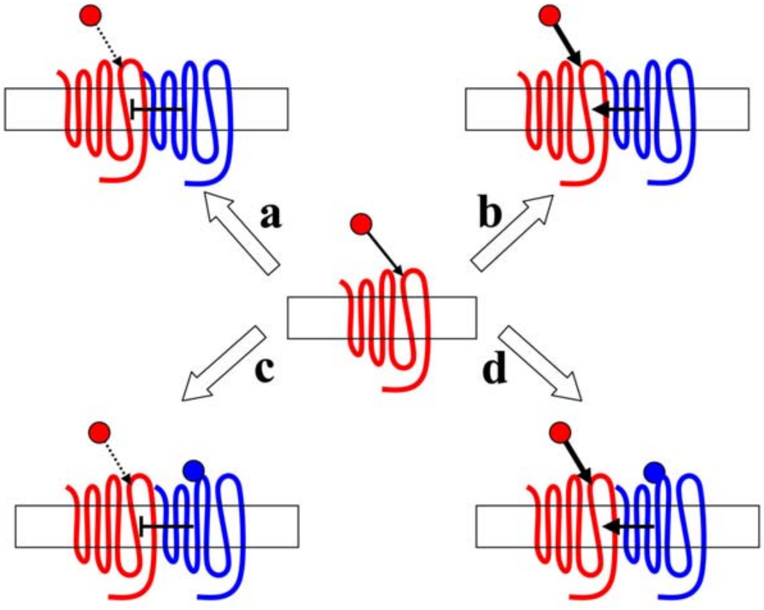

**FIGURE 3.** Intramolecular interactions in the receptor heterodimer. The affinity of a given ligand for its receptor (central drawing) may decrease (a) or increase (b) when the receptor forms heterodimers and also may be decreased (c) or increased (d) when the receptor partner (in the heterodimer) is occupied by the same (for isoreceptors) or another ligand.

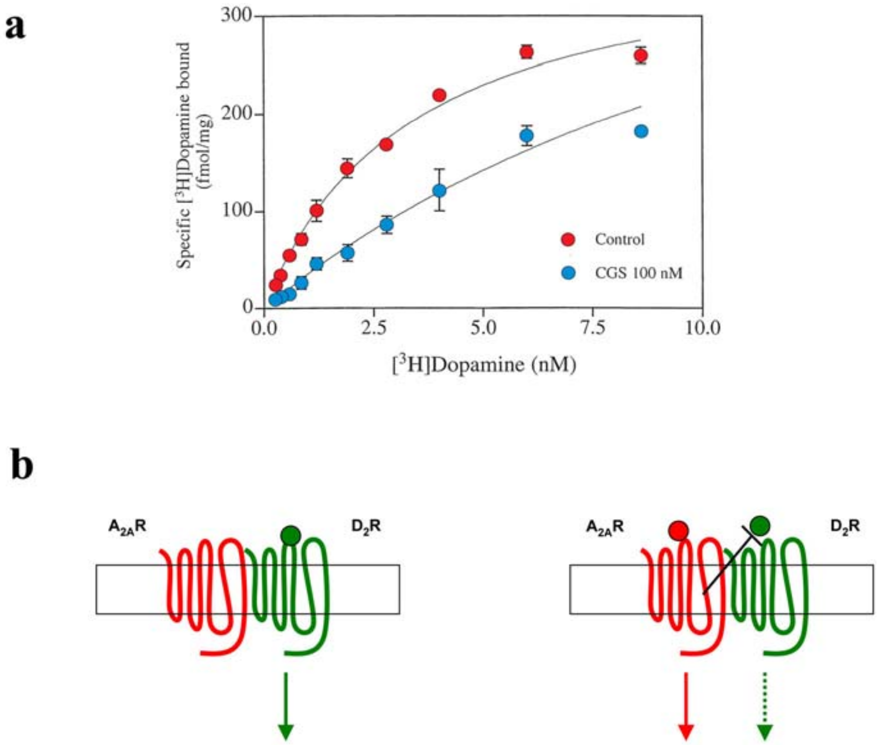

**FIGURE 4.** Biochemical fingerprint of the adenosine $A_{2A}$-dopamine $D_2$ receptor heterodimer. (a) Saturation curves of specific binding of [$^3$H]dopamine in membrane preparations from CHO cells stably cotransfected with adenosine $A_{2A}$ and dopamine $D_2$ receptors in the presence and absence of the $A_{2A}$ receptor agonist CGS 21680 (modified from Franco et al.[36]). The results show a strong modulatory effect of $A_{2A}$ receptors on the affinity of $D_2$ receptors for dopamine ($K_D$ values in the absence and presence of CGS 21680 were 3.3 and 15.3 n*M*, respectively; results represent means ± SD of triplicate data). (b) Scheme of the intramembrane interaction between $A_{2A}$ and $D_2$ receptors in the $A_{2A}$-$D_2$ receptor heterodimer. Stimulation of $A_{2A}$ receptors decreases the ability of dopamine to stimulate $D_2$ receptors.

Fingerprints for heterodimers can also be detected by changes in signaling triggered by a given neurotransmitter-drug. As a recent example, George's group[27] has identified dopamine $D_1$-$D_2$ receptor heterodimers in the brain that are selectively coupled to $G_{q/11}$ proteins, and that require agonist binding to both receptors for $G_{q/11}$ protein activation and intracellular calcium release. The authors showed that $D_2$ receptor normally couples to $G_{i\text{-}o}$ proteins, but in the $D_1$-$D_2$ receptor heterodimer, it switches to $G_{q/11}$ when the $D_1$ receptor is coactivated. In this way, the $D_1$-$D_2$ receptor heterodimer provides a selective mechanism by which dopamine activates a phospholipase C–mediated calcium signaling[27] (Fig. 5).

Another recent example of changes in signaling is the recently described adenosine $A_{2A}$-cannabinoid $CB_1$ receptor heterodimer. The mechanism of action responsible for the motor-depressant effects of cannabinoids, which operate through centrally expressed cannabinoid $CB_1$ receptors, is still a matter of debate. Carriba et al.[28] have reported that $CB_1$ and adenosine $A_{2A}$ receptors form heteromeric complexes in cotransfected HEK-293T cells and rat striatum, where they colocalize in fibrillar structures. In a human neuroblastoma cell line, $CB_1$ receptor signaling was found to be completely dependent on $A_{2A}$ receptor activation. Accordingly, blockade of $A_{2A}$ receptors counteracted the motor-depressant effects produced by the intrastriatal administration of a cannabinoid $CB_1$ receptor agonist. These biochemical and behavioral findings demonstrate that the profound motor effects of cannabinoids depend on physical and functional interactions between striatal $A_{2A}$ and $CB_1$ receptors[28].

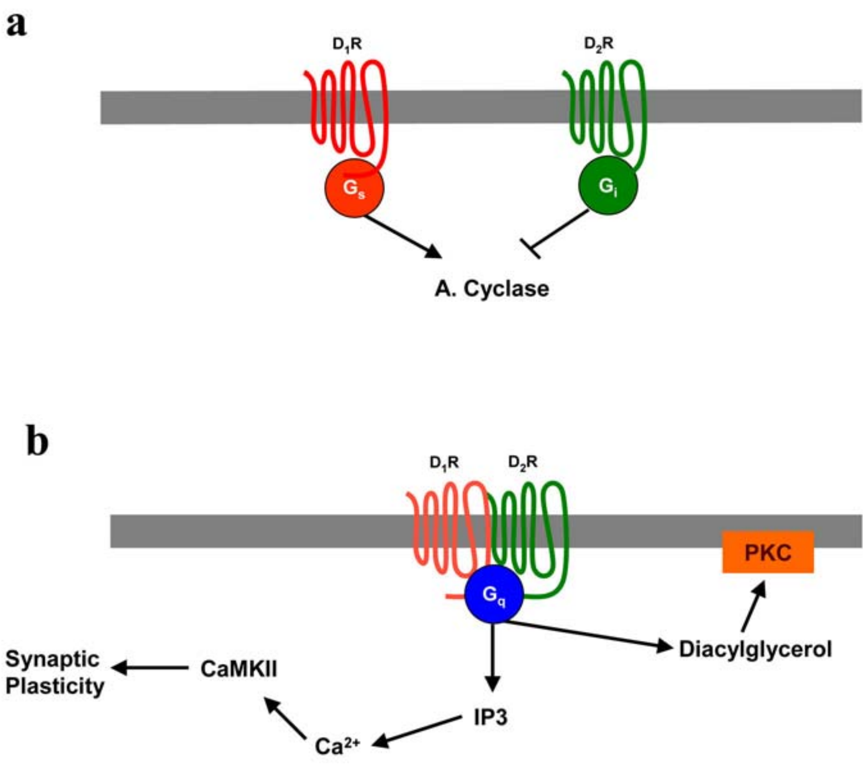

**FIGURE 5.** Biochemical fingerprint of the dopamine $D_1$-$D_2$ receptor heterodimer. (a) When not forming heterodimers, $D_1$ receptor couples to $G_s$ proteins, whereas $D_2$ receptor couples to $G_i$ proteins. (b) $G_q$ selectively couples to $D_1$-$D_2$ receptor heterodimers, which in turns leads to a dopamine- and calcium-dependent activation of calcium calmodulin kinases, with possible implications for synaptic plasticity[27].

Also recently, Rozenfeld and Devi[29] have shown that heterodimerization of μ-opioid receptors (μOR) with δ-opioid receptors (δOR) leads to a constitutive recruitment of β-arrestin2 to the receptor complex, resulting in changes in the spatiotemporal regulation of ERK1/2 signaling. In the naive state, μOR-δOR heterodimers are in a conformation conducive to β-arrestin–mediated signaling. Destabilization of this conformation by cotreatment with μOR and δOR ligands leads to a switch to a non-β-arrestin–mediated signaling. Taken together, these results show that μOR-δOR heterodimers, by differentially recruiting β-arrestin, modulate the spatiotemporal dynamics of opioid receptor signaling[29].

## RECEPTOR HETERODIMERS AS TARGETS FOR CNS-ACTING DRUGS

Identification of dimers-oligomers of GPCRs in the CNS opens a new way to look into the effect of neurotransmitter-neuromodulators. Opioid receptor heterodimers constitute a clear example. In the opioid receptor family, δOR have been shown to interact *in vitro* with both κ- and μOR to form heterodimers, and this leads to altered pharmacological properties[30,31,32,33]. The κOR-δOR heterodimers were found to have reduced affinities for selective κ and δ ligands[30]. Devi's group has shown that potentiation of μOR agonist binding by a δOR antagonist leads to increased antinociceptive morphine response using a classical analgesia test[33].

An emerging notion with GPCR dimerization is that properties enabled by the interaction of two protomers could be used to design new classes of compounds with more efficacious therapeutic properties[34]. Waldhoer et al.[35] have shown that the opioid agonist ligand 6'-guanidinonaltrindole (6'-GNTI) has the unique property of selectively activating only opioid receptor heterodimers, but not homomers. Importantly, 6'-GNTI is an analgesic, thereby demonstrating that opioid receptor heterodimers are indeed functionally relevant *in vivo*. However, 6'-GNTI induces analgesia only when it is administered in the spinal cord, but not in the brain, suggesting that the organization of heterodimers is tissue specific. This study demonstrates a proof of concept for tissue-selective drug targeting based on GPCR heterodimerization[36]. Importantly, targeting opioid heterodimers could provide an approach toward the design of analgesic drugs with reduced side effects[35].

## CONCLUSIONS

Receptor dimers (homo or hetero) are the actual targets of neurotransmitters, neuromodulators, or compounds-drugs binding to the orthosteric center of GPCRs. The occurrence of receptor dimers leads to the concept of cooperativity in the binding of the second molecule to the second receptor in the dimer. If the binding is improved after the binding of the first ligand positive cooperativity occurs; otherwise, negative cooperativity is displayed when representing data from binding assays. Any other molecule binding to nonorthosteric centers in the dimer becomes an allosteric effector, which includes G proteins, or natural or synthetic small molecules. Cooperativity and allosterism must be used carefully when referring to receptor dimers. Apart from affinities of the binding to the two receptors in the dimer, a new parameter, the dimer cooperativity index, has been devised that quantitates the degree of intramolecular interaction happening on ligand binding. Interestingly, allosterism can take advantage of the dimer-cooperativity index since, quite often, allosteric regulators modify cooperativity. It is also important to note that coactivation of receptors in a heterodimer usually changes the signaling pathway triggered by a neurotransmitter-drug and this is not only important for physiology, but also for therapeutic-pharmaceutical developments. A correct nomenclature, clear concepts, and new tools (as the dimer cooperativity index) are not only important to understand the physiological role of GPCRs, but also to characterize them and even to detect them in natural tissues. In fact, the most effective way to detect dimers in the CNS is to look for a dimer fingerprint. Apart from the examples given in other sections of this issue, it should be noted that identification of a biochemical fingerprint can be a relatively easy task for a number of heterodimers. Finally, it should be noted that compounds that act specifically on receptor heterodimers could represent the next generation of neuropsychotropic drugs.

## ACKNOWLEDGMENTS

Work supported by grants from the Spanish Ministerio de Ciencia y Tecnología SAF2006-05481 to R.F. SAF2005-00170 to E.I.C; Grant 060110 from Fundació La Marató de TV3 to E.I.C. and the NIDA IRP funds.
